# A Systematic Review of Workplace Physical Activity Coaching

**DOI:** 10.1007/s10926-023-10093-8

**Published:** 2023-02-27

**Authors:** A. Gawlik, J. Lüdemann, A. Neuhausen, C. Zepp, F. Vitinius, J. Kleinert

**Affiliations:** 1 Department of Health and Social Psychology, Institute of Psychology, German Sport University Cologne, Cologne, Germany; 2 Department of Psychosomatics and Psychotherapy, Faculty of Medicine, University Hospital Cologne, University of Cologne, Cologne, Germany

**Keywords:** Physical activity, Coaching, Workplace, Health intervention, Sedentary behavior

## Abstract

*Aim* Studies show that about 60 min of moderate physical activity (PA) per day compensate for sitting all day at work. However, the workplace offers an ideal setting for health-promoting interventions such as PA coaching as a person-centered intervention aimed at achieving lasting health behavior changes. Given a good evidence base of health coaching studies in general, this systematic review aims to provide an overview of workplace PA coaching interventions. *Methods* This review was conducted according to PRISMA guidelines. Studies published up to July 2021 were considered based on the following inclusion criteria: (1) longitudinal intervention studies, (2) analysis of PA at work, (3) sedentary employees, (4) PA coaching in the workplace as intervention, (5) increasing workplace PA. *Results* Of 4323 studies found, 14 studies with 17 interventions met inclusion criteria. All 17 interventions indicated an increase in at least one PA outcome. Twelve interventions indicated significant improvements in at least one workplace or total PA outcome. There is a high variation within the different coaching parameters, such as behavior change techniques and communication channels. The study quality showed a moderate to high risk of bias. *Conclusions* The majority of interventions provided evidence for the effectiveness of workplace PA coaching. Nevertheless, the results are inconclusive with regard to the variety of coaching parameters and thus no general statement can be made about the effectiveness of individual parameters. However, this variety of parameters also leads to a high degree of individualization of workplace PA coaching interventions to increase PA for different groups of employees and different types of workplaces.

**Supplementary Information:**

The online version of this article contains supplementary material available 10.1007/s10926-023-10093-8.

## Introduction

More than half of the adult population are physically active for less than two and a half hours per week and hence do not meet the core aspect of the WHO recommendations for physical activity (PA; [1–3]). Despite well-publicized national health campaigns and a large choice of recreational activities, this statistic is surprising. The extensive positive effects are multifaceted and refer to both physical as well as psychological improvements at any age [4]. Physical improvements relate to, for example, reduced risks for weight gain and overweight/obesity, type 2 diabetes, cardiovascular diseases, stroke, age-related illnesses such as dementia and Alzheimer’s disease, and several types of cancer [5–9]. Psychological improvements include the reduction of symptoms of depression and anxiety, improvement of mood, and stress management [10, 11].

One method to increase PA behavior is physical activity coaching (PA coaching). Coaching to change a health behavior, in this case PA, is a collaborative patient- or person-centered approach to empower individuals to take responsibility for their PA behavior and to facilitate their achievement of PA-related goals in order to change their PA behavior permanently [12–14]. PA coaching can be installed in many different settings, such as schools, health care organizations, community centers, recreational facilities, and workplaces [15, 16].

The workplace, in particular, is an ideal setting for the implementation of PA coaching. It can overcome commonly cited barriers, such as lack of time, and provides access to a broad and diverse section of society [17–19]. Especially, people with particular health risks are easier to reach in the workplace than in the leisure time, where there are often major barriers to accessing health programs. Other advantages of the workplace as a health-promoting setting include the "convenient place and time," as well as the possibility of "paid time off" while being physically active; [20]). In addition, company leadership has a responsibility to ensure and promote the health of their employees [21]; conversely, they also benefit greatly from the long-term good health of their employees in terms of lower health care costs and added working time [22–24]. However, in addition to these benefits, the workplace also presents some issues that need to be addressed, such as relatively little time available for health programs and organizational challenges.

PA coaching interventions are characterized by different parameters, such as the time scope or organization of coaching. Time scope includes, for example, the duration of the intervention (short-term vs. long-term; [13] and the frequency and duration of the coaching interactions (speed coaching vs. longer conversation; [13]). Organization of coaching includes, for example, the communication channel (e.g., in person, telephone, web [25–27] and additional voluntary interventions including environmental modifications (e.g., walking tracks outside a company; [28]). Further parameters comprise the underlying theory (e.g., self-determination theory, SDT, [29]; transtheoretical model of change, TTM, [30]; social cognitive theory, SCT, [31]) and the question of which and how many behavior change techniques (BCTs) are applied [32]. The role of the coach in PA coaching is active listening, supporting, motivating [25], and using motivational strategies to change health behaviors [33], such as goal setting, social support, and barrier management [13, 30].

Despite the wide variety of compositions of PA coaching interventions, numerous studies showed positive effects on the PA behavior for patients with different chronic conditions, for inactive people, and in different settings [34–38]. In addition to these primary studies, recent reviews also summarized the effects of PA coaching, showing positive effects on the PA behavior in different target groups, for example, inactive adults [39], patients with chronic diseases [40], and the elderly [41, 42]. So far, however, there are only reviews that summarize general workplace physical activity promotion interventions (e.g., [17, 43]). Likewise, some workplace PA coaching interventions showed positive effects according to Dugdill et al. [17]. Although there is evidence for PA coaching interventions in a variety of settings, and likewise in the workplace, a review of studies summarizing solely PA coaching in the workplace is yet lacking.

Considering the increasing importance of PA promotion, the known positive effects of PA on health, as well as the positive effects of coaching, it is important to understand how workplace PA can be promoted in workplace coaching programs. Therefore, the purpose of this review is to summarize existing coaching interventions promoting workplace PA in order to provide an up-to-date overview of intervention studies. Specific objectives include (1) describing the characteristics of these interventions (e.g., time scope of coaching, organization of coaching, theoretical foundation, applied BCTs) and (2) determining whether these interventions have a positive impact on PA.

## Methods

This systematic review was conducted in line with PRISMA guidelines [44]. The protocol of the study is registered with the PROSPERO database and can be accessed under reference number CRD42021256548.

### Inclusion Criteria

To be included in this review, studies had to be longitudinal intervention studies. For this review, the intervention, PA coaching in the workplace, was defined as personalized, person-centered, interactive PA coaching (within the institution/company) that is either web-based, telephone-based, mobile-based, or in person. When it comes to e-coaches (web-based/mobile-based), this review included interventions that considered e-coaching systems as a computerized part of a system that uses an artificial entity to observe, learn from, and support user behavior in a proactive collaboration applying planning and goal-related techniques [45]. There had to be at least one interaction between coach and coachee (coached employee) and it could be either individual or group coaching. Interventions had to aim at increasing health-enhancing PA in the workplace (plus optional leisure time PA, subjectively and/or objectively assessed). Health-enhancing PA was defined as “any form of physical activity that benefits health and functional capacity without undue harm and risk” [46]. Furthermore, interventions had to be addressed toward sedentary employees. Sedentary work was defined as "involving lifting no more than 10 pounds at a time and occasionally lifting and carrying articles like, docket files, ledgers, and small tools" (The United States Social Security Administration 2012). The reason for including sedentary employees is that prolonged sitting at work in particular can pose a health risk [47]. Studies that did not meet the inclusion criteria were excluded from this review.

There were no restrictions on the basis of sample size, participant characteristics (e.g., age, gender), type of PA coaching intervention, study length, duration of follow-up, or publication date. Randomized, controlled, and quasi-experimental studies, as well as pilot studies were included, as a pure randomized controlled trial (RCT) design is not always possible in a workplace setting. Similarly, multicomponent health promotion interventions with the main aim of improving general health were included if they provided an outcome measure that focused specifically on employees’ PA.

### Literature Search

The literature search was conducted using the following electronic databases: PsycINFO, PsycArticles, PSYNDEX, Web of Science, SocIndex, and MEDLINE. A broad search strategy was elaborated using a combination of specified search terms (Table 1). Peer-reviewed studies published in English and German up to July 2021 were retrieved. Two review authors independently reviewed the titles and abstracts of all potentially relevant articles for eligibility. Either articles were confidently included, confidently excluded, or this decision was made after full-text screening when still uncertain. Any disagreements between the two were resolved through discussions involving a third person.

**Table 1:** Literature search strategy

Keyword combination	
1. (work$ or occupation$ or labour$ or employ$ or job$) ti, ab 2. (coach$ or counsel$ or train$ or health-coach$ or program$) ti, ab 3. (physical activ$ or health-enhanc$ or HEPA or sport$ or exercis$) ti, ab 4. (sedentary$ or sitt$ or inactiv* or desk-bound or stationary) ti, ab 5. 1 AND 2 AND 3 AND 4 6. remove duplicates from 5	

*ti* Title, *ab* abstract. These words had to appear in the title and abstract

### Data Extraction

Two review authors extracted the data independently and merged them afterwards. Likewise, when discrepancies were identified during this process, they were resolved in a conversation with a third person. The extracted data included the author(s), year of data collection, country/region of data collection, type of study, sample size, age, gender, response rate, professional sector, other behaviors potentially addressed in coaching, duration of intervention, frequency and duration of coaching interactions, group/individual intervention, communication channel, voluntary interventions in addition to coaching, underlying theory/model, number and name of applied BCTs, PA outcomes (workplace, leisure time, transport), and measurements used to assess the PA outcome(s).^1^ In addition to these categories reported in the protocol, the goals of the interventions as well as the numbers and types of the control group/further intervention groups were also reported. Furthermore, the material provided during coaching, the PA outcomes, and the type of coach (person/e-coach) were added. Due to lack of data, the type of sampling as written in the protocol was not considered for this review.

### Quality Assessment

The methodological quality of each study was assessed independently by two review authors using the revised and validated Cochrane risk of bias tools for randomized trials (RoB 2, [48]) and for non-randomized trials (ROBINS-I, [49]). When discrepancies were identified during this process, they were resolved in a conversation with a third person. Additionally, this review used the taxonomy of BCTs developed by Michie et al. [32] to derive and better compare the specific BCTs used in the coaching interventions promoting PA. Due to assumed heterogeneous study designs, no overall meta-analytical effect sizes were analyzed in this paper.

## Results

**Fig. 1:**
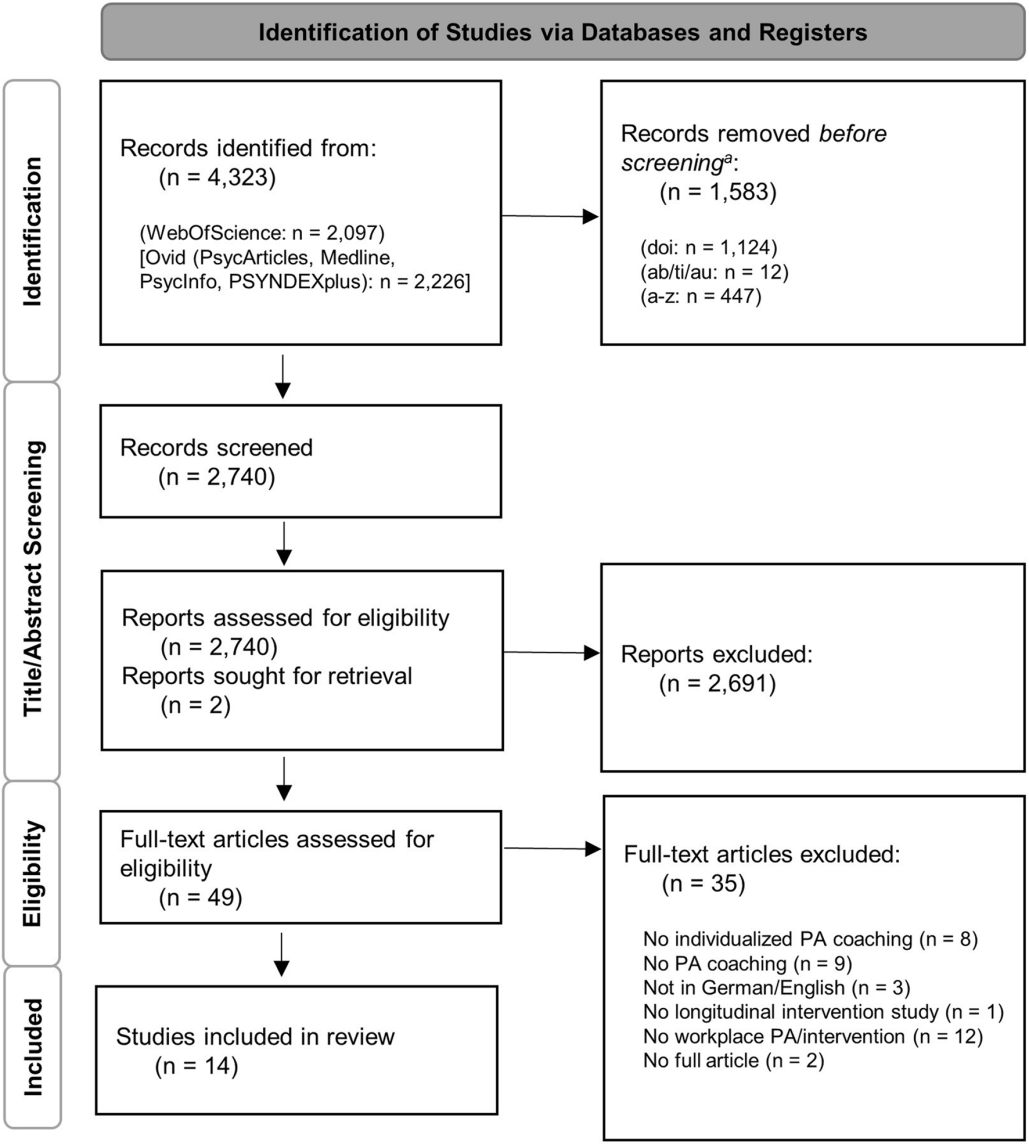
Flow Chart of the study selection process. Note. ^a^Records were manually removed by digital object identifier (doi), abstract (ab), title (ti), author (au)

The initial computerized search found 4323 publications (Fig. 1). Computerized duplication removal of several factors (using doi, filtering by abstract, title, and authors, and sorting from a to z) resulted in 2740 publications. The authors of two publications of which only the abstract was available online were contacted but without success. After title and abstract screening of the 2740 studies by two review authors, 2691 studies were further excluded. The remaining 49 studies were reviewed for full-text screening. After full-text screening, 35 studies were excluded. Reasons for exclusion included, for example, lack of individualization in PA coaching (n = 8), no PA components in coaching (n = 9) or no longitudinal intervention study (n = 1). Ultimately, 14 studies were selected for this review (detailed information in Table 2 and Table 3).

**Table 2:** Study characteristics and parameters of workplace PA coaching

No	Author Year Continent	Sample characteristics (sample size, age (M +SD), gender, professional sector, response rate)	Study design (design, control group, follow up)	Goals	Time scope of coaching (duration of intervention, total number and duration of interactions, frequency)	Organization of coaching (intervention setting, communication channel, coaching material, additional voluntary interventions)	Theory	BCTs	Coach	Outcomes (plus measurement)
				Behavior addressed in coaching in included IG						
1	Arrogi 2017 Europe	246 41 years (8.8) 76% female, 24% male pharma 91%	Non-RCT Passive CG Pre/post/follow-up after 6 months	Increase in PA None	3 months 9 interactions (6 individual ftf sessions at 60 min. + 3 email/phone calls) One interaction every 3 weeks	Individual Ftf + email/phone Information leaflet for maintenance Offer of an individualized PA program	SDT	6 Goal setting, action planning, social support, self-monitoring, feedback, problem solving	Trained professional scientific coach	PA intensity (METs), daily step count (weekdays, weekend days, average days) (accelerometer: SWA Pro3), daily min. of MVPA (questionnaire: IPAQ)
2	Boerema 2019 Europe	15 58.93 years (5.4) 53% female, 47% male university 93%	Non-RCT No CG Pre/post/no follow-up	Increase in PA & decrease in SEB SEB	2 weeks 98 interactions (6 feedback messages per day (= 84) + 1 short questionnaire per day (= 14))	Individual App	–	2 Feedback, prompts/cues	E-coach	Mean PA intensity of counts per min. per day, PA min./hour of wear time (accelerometer: ProMove 3D activity sensor)
3	Bort-Roig 2020 Europe	89 45 years (9) 82% female, 18% male hospital 42%	RCT Active CG with tracking app Pre/post/no follow-up	Increase in PA & decrease in SEB SEB	13 weeks ~ 99 interactions (1 feedback message per day (= 91) + further weekly and fortnight messages)	Individual App	–	5 Feedback, self-monitoring, behavior substitution, prompts/cues, goal setting	E-coach	Stepping time (hours), light intensity PA (hours), MVPA (min.) (accelerometer: activPAL3TM)
4	Chae 2015 Asia	70 38.31 years (8.46) 50% female, 50% male airline 55.7%	Non-RCT No CG Pre/post/no follow-up	Increase in PA None	8 weeks 4 interactions (1 group session at 30 min. + 3 individual sessions) 2 × in week 1, 1 × week in 4, 1 × in week 8	Group + individual Ftf Booklet	SCT	5 Goal setting, self-monitoring, feedback, information about health consequences, social support	Trained professional health care coach	Daily step count (pedometer: Yamax CW700/701)
5	Gilson 2017 Australia	6 47.5 years (9.8) 0% female, 100% male trucking industry 73.1%	Non-RCT No CG Pre/post/follow-up after 8 weeks	Increase in PA & improvement of dietary behavior Dietary behavior	20 weeks 7 interactions (2 ftf group sessions + 5 individualized feedbacks) 1 × in week 0, 2 × in week 1 + since then every 4^th^ week	Group + individual Ftf + app	–	9 Goal setting, action planning, behavior substitution, habit formation, information about health consequences, adding objects to the environment, self-monitoring, feedback, rewards/ incentives	Trained professional scientific coach	Time spent walking or running, proportions of PA in % (walking + running combined) (working time, workday non-working time, non-workday) (accelerometer: GENEActiv wrist)
6	Lee 2019 Asia	41 37.68 years (9.31) X manufacturing 100%	Non-RCT Active CG with tracker Pre/post/no follow-up	Increase in PA None	12 weeks 102 interactions (1 ftf sessions at 5 min. biweekly (= 6) + 1 message per day (= 84) + 1 weekly motivational messages (= 12)	Individual Ftf + app Workbook Posters + banners in cafeterias/gates	–	7 Feedback, self-monitoring, goal setting, action planning, problem solving, prompts/cues, biofeedback	Not reported + e-coach	Daily walking, step count (accelerometer: Fitbit Charger HR), PA behavior (questionnaire: PA subscale of HPLP-II)
7	Nooijen 2020 Europe	83 41 years (9) 80% female, 20% male product & service producing companies 82%	RCT Passive waitlist CG + IG with SEB intervention Pre/post/no follow-up	Increase in PA & decrease in SEB (different IGs) & examination of mental health None	24 weeks 5 interactions (3 individual ftf sessions at 45–60 min. + 2 group sessions at 90 min.) Spread over 6 months	Group + individual Ftf Access to a gym, exercise sessions, lunch walks, provision of company bikes, sit-stand desks, team leaders	–	2 Feedback, social support	Trained professional health care coach	Time spent in MVPA (and light, moderate, vigorous), quantification of time walking (% of wear time) (all days/weekdays only) (accelerometer: Actigraph GT3X)
8	Opdenacker a 2008 Europe	45 38.8 years (11.4) X university 73%	RCT Other IG with telephone coaching (Opdenacker b.) Pre/post/no follow-up	Increase in PA & examination of mental health None	12 weeks 5 interactions (5 individual ftf sessions) 2 × in month 1, 1 × in month 2, 1 × in month 3	Individual Ftf Brochure Walk plotting, cycling routes around campus, placing prompts to use stairs, "start-to-run" during lunch time, sports promoters	–	5 Information about health consequences, problem solving, goal setting, self-monitoring, social support	Trained professional scientific coach	Min. per week of moderate + vigorous PA in 4 domains: job-related PA, active transportation, PA during housework + garden activities, and leisure time (questionnaire: IPAQ)
9	Opdenacker b Europe	45 39.9 years (9.9) X university 73%	RCT Other IG with ftf coaching (Opdenacker a.) Pre/post/no follow-up	Increase in PA & examination of mental health None	12 weeks 5 interactions (1 individual ftf session + 4 phone calls) 2 × in month 1, 1 × month 2, 1 × month 3	Individual Ftf + phone Brochure Walk plotting, cycling routes around campus, placing prompts to use stairs, "start-to-run" during lunch time, sports promoters	–	5 Information about health consequences, problem solving, goal setting, self-monitoring, social support	Trained professional scientific coach	Min. per week of moderate + vigorous PA in 4 domains: job-related PA, active transportation, PA during housework + garden activities, and leisure time (questionnaire: IPAQ)
10	Poirier 2016 North America	133 40.3 years (11.4) 62% female, 38% male multinational health care company 80.5%	RCT Passive CG Pre/post/no follow-up	Increase in PA None	6 weeks Up to 168 interactions (Up to 4 messages per day, on demand)	Individual Messaging (email/SMS or web-based) Website	–	5 Goal setting, self-monitoring, feedback, rewards/incentives, social support	E-coach	Average step count per day (accelerometer: Pebble +)
11	Proper 2003 Europe	131 43.8 years (8.3) 26% female, 74% male municipal services 84%	RCT Active CG with information Pre/post/no follow-up	Increase in PA & examination of health-related fitness and health Diet, stress, smoking behavior	36 weeks 7 interactions (7 individual ftf sessions at 20 min.)	Individual Ftf Written information	TTM	1 Action planning	Trained professional health care coach	Total energy expenditure, days of moderate intensity PA ~ 30 min. a day, PA during leisure time, level of activities during sport (questionnaire: questions about physically active days)
12	Purath 2004 NI	134 44.6 years (9.9) 100% female, 0% male university 90%	RCT Active CG with non-tailored coaching Pre/post/no follow-up	Increase in PA None	6 weeks 2 interactions (1 individual ftf session at 3–5 min. + 1 booster phone call) 1 × in week 1, 1 × in week 3	Individual Ftf + phone Optional pamphlets via email	TTM	4 Information about health consequences OR goal setting, behavioral contract PLUS goal setting OR feedback AND problem solving	Trained professional health care coach	Number of flights of stairs/day, number of blocks walked/day, hours of vigorous + moderate PA on weekdays, hours of vigorous + moderate weekend PA, min. walked per week: for exercise, on errands, during breaks or lunch, to work or school, total walking (questionnaires: Paffenbarger PA questions, PACE© walking questions)
13	Ribeiro a 2014 South America	53 45 years (3) 100% female, 0% male university hospital 87%	RCT Other IG with group coaching (Ribeiro b.) + passive CG + CG with aerobic training Pre/post/follow-up after 6 months	Increase in PA & reduction of anthropometric measures None	12 weeks 3 interactions (3 individual ftf sessions at 15 min.)	Individual Ftf Booklet + step diary	–	4 Information about health consequences, instruction on how to perform the behavior, self-monitoring, goal setting	Trained professional health care coach	Total number of steps, steps performed at moderate-intensity levels (frequency > 110 steps per min.) (pedometer: Digiwalker, Power Walker Model 610)
14	Ribeiro b 2014 South America	48 45 (3) 100% female, 0% male university hospital 66.7%	RCT Other IG with individual coaching (Ribeiro a.) + passive CG + CG with aerobic training Pre/post/follow-up after 6 months	Increase in PA & reduction of anthropometric measures None	12 weeks 8 interactions (8 group sessions (with 12p) at 60 min.) 1 × with 1-week interval in week 1–6, 1 × with 2-week interval in week 7 and 8	Group Ftf Booklet + step diary	–	5 Information about health consequences, problem solving, goal setting, social support, self-monitoring	Trained professional health care coach	Total number of steps, steps performed at moderate intensity levels (frequency > 110 steps per min.) (pedometer: Digiwalker, Power Walker Model 610)
15	Sternfeld 2009 North America	195 45.3 years (10.2) 76% female, 24% male health care delivery company 50.2%	RCT 2 other IGs (fat, fruits/veg.) + CG (individualized feedback on dietary & PA behavior) Pre/post/follow-up after 4 months	Increase in PA & improvement of dietary behavior (different groups) None	16 weeks 12 interactions (12 emails) Biweekly for 2 months, then every other week for 2 months	Individual Messaging (email) Interactive informative website	TTM	6 Goal setting, information about health consequences, self-monitoring, problem solving, behavior substitution, discrepancy between current behavior and goal	Not reported	Total activity in MET-min. per week, moderate PA in min. per week, vigorous PA in min. per week, walking in min. per week (questionnaire: PAQ)
16	Tucker a 2016 NI	27 43 years (12.4) 100% female, 0% male ambulatory clinic nursing 85.7%	RCT Cross-over (Tucker a. + b.) Pre/post/follow-up after 6 months	Increase in PA & decrease in SEB & improvement of body composition None	12 weeks 84–168 interactions (1–2 messages per day)	Individual Messaging (SMS) EARLY TEXTING Workstation treadmill, Wii™ video game system, stair climbing, walking meetings	–	2 Goal setting, monitoring of outcome(s) of behavior without feedback	Trained professional scientific coach	Moderate activity, vigorous activity, active energy expenditure (METs), steps per hour of time awake (averages per day, % time) (accelerometer: SWA Mini)
17	Tucker b 2016 NI	13 42.2 years (12) 100% female, 0% male ambulatory clinic nursing 85.7%	RCT Cross-over (Tucker a. + b.) Pre/post/follow-up after 6 months	Increase in PA & decrease in SEB & improvement of body composition None	12 weeks 84–168 interactions (1–2 messages per day)	Individual Messaging (SMS) LATE TEXTING Workstation treadmill, Wii™ video game system, stair climbing, walking meetings	–	2 Goal setting, monitoring of outcome(s) of behavior without feedback	Trained professional scientific coach	Moderate activity, vigorous activity, active energy expenditure (METs), steps per hour of time awake (averages per day, % time) (accelerometer: SWA mini)

*BCT* behavior change technique, *CG* control group, *ftf* face-to-face, *HPLP-II* Health-Promoting Lifestyle Profile II questionnaire, *HR* heart rate, *IG* intervention group, *IPAQ* International Physical Activity Questionnaire, *M* mean, *MET* metabolic equivalent of task, *MVPA* moderate-to-vigorous physical activity, *NI* no information, *PA* physical activity, *PAQ* physical activity questionnaire, *professional coach* holding a degree + trained in coaching, *RCT* randomized controlled trial, *SCT* social cognitive theory, *SD* standard deviation, *SDT* self-determination theory, *SEB* sedentary behavior, *SWA* Sensewear® Armband, *TTM* transtheoretical model

**Table 3:**
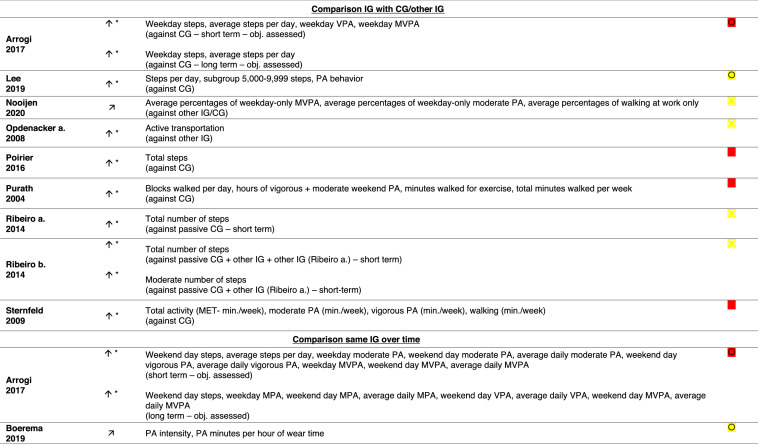

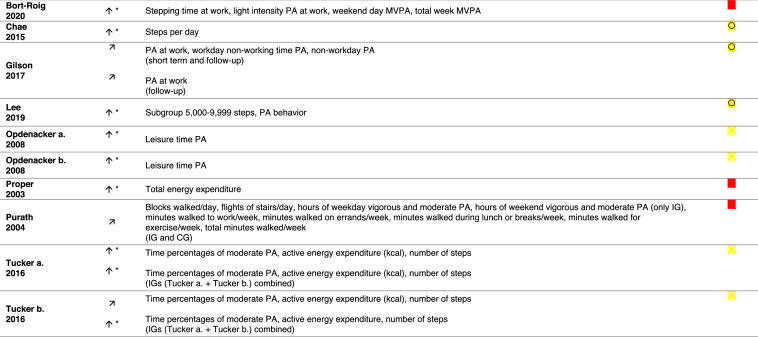
Summary of intervention effects on PA and risk of bias assessment

CG = control group, IG = intervention group, MET = metabolic equivalent of task, MVPA = moderate-to-vigorous physical activity, obj. = objectively (measured), PA = physical activity, VPA = vigorous physical activity, * = significant increase in this/these PA outcome(s), = increase in this/these PA outcome(s),  = moderate risk of bias (ROBINS-I),  = some concerns in risk of bias (RoB 2),  = critical risk of bias (ROBINS-I), = high risk of bias (RoB 2)

### Sample Characteristics

The total sample size amounted to N = 1394 and ranged from 13 to 246 (*M* = 82) between the 14 studies^2^ examined. Seven of the 14 studies had a majority of female participants (> 70%) (Table 2; interventions 1, 3, 7, 12–17), out of those, three studies were conducted exclusively with female subjects (Table 2; interventions 12–14, 16, 17). There was only one study with exclusively male participants (Table 2, intervention 5). Two studies provided no indication of gender (Table 2; interventions 6, 8, 9). The participants’ median age ranged from 38.3 to 58.9 years. Overall, in 11 studies the participants’ median age was between 40 and 50 years (Table 2; interventions 1, 2, 3, 5, 7, 10–17). Employees were recruited from health care organizations (Table 2; interventions 1, 3, 10, 13–17), universities (Table 2; interventions 2, 8, 9, 12), the production sector (Table 2; interventions 6, 7), the transportation sector (Table 2; interventions 4, 5), as well as “other services” [municipal services (Table 2, intervention 11)]. Response rates at the last measurement point ranged between 42 and 100% (*M* = 77%). Eleven studies had a response rate > 70% (Table 2; interventions 1, 2, 5–13, 16, 17).

### Study Design

Nine of the 14 studies were randomized controlled trials (RCTs) (Table 2; interventions 3, 7–17). The remaining studies were non-randomized trials (Table 2; interventions 1, 2, 4–6).

Among the RCTs, different control group designs were used [passive control groups (Table 2; interventions 7, 10)], additional intervention group(s) (Table 2; interventions 7, 8, 9, 13–17), active control group (Table 2; interventions 3, 11, 12). The non-RCTs also included different groups in the studies (no control group (Table 2; interventions 2, 4, 5), an active control group (Table 2, intervention 6), a passive control group (Table 2, intervention 1)].

Regarding their follow-up measurement, nine studies measured effects before and after the intervention (Table 2; interventions 2–4, 6–12) with five studies applying a follow-up measurement [after eight weeks (Table 2, intervention 5), after four months (Table 2, intervention 15), after 6 months (Table 2; interventions 1, 13, 14, 16, 17)].

### Goals and Behaviors Addressed in Coaching

In terms of goals, increasing PA is the only objective reported in five of the 17 interventions^2^ (Table 2; interventions 1, 4, 6, 10, 12). Nine interventions reported the evaluation of other variables and additional goals, such as the reduction of sedentary (Table 2; interventions 2, 3, 7, 16, 17) and dietary behavior (Table 2, intervention 5) or the examination of mental health (Table 2; interventions 7–9, 11).

In terms of behaviors addressed in coaching, 13 interventions solely targeted PA behavior (Table 2; interventions 1, 4, 6–10, 12, 13–17). The other four addressed additional behaviors, such as sedentary and dietary, stress, and smoking behavior in their coaching (Table 2; interventions 2, 3, 5, 11).

### Time Scope of Coaching

The scope of coaching, including the duration of the intervention, the total number of interactions, the duration and frequency of the interactions, was very heterogeneous. The duration of PA coaching ranged from two to 36 weeks with the most common frequency being 12 weeks (Table 2; interventions 1, 6, 8, 9, 13, 14, 16, 17). The total number of interactions ranged from two to 168 interactions. Eleven interventions had two to 12 interactions (Table 2; interventions 1, 4, 5, 7–9, 11, 12–15), and six interventions had more than 84 to 168 interactions (Table 2; interventions 2, 3, 6, 10, 16, 17). In three of the 17 interventions, wide ranges of interactions between e-coach and coachee were reported, because in these cases the coachees themselves determined how often they “approached” their e-coach (Table 2; interventions 10, 16, 17). To ensure comparability, the total highest number of interactions was therefore reported in this review. The frequency of coaching interactions (meaning how often interactions between coach and coachee occurred during the duration of the intervention) was regular (daily, biweekly, triweekly) in eight of the included interventions (Table 2; interventions 1, 2, 3, 6, 10, 15 at the beginning, 16, 17). In seven interventions, interactions were more frequent in the initial phase and then subsided with the intervention period (Table 2; interventions 4, 5, 8, 9, 12, 14, 15 at the end). Three interventions did not provide any information on the frequency of coaching contacts (Table 2; interventions 7, 11, 13).

### Organization of Coaching

The organization of coaching, which involves the setting, communication channel, additional coaching materials, or additional voluntary interventions to increase workplace PA, also varied widely across all interventions. Thirteen of the 17 interventions included were individual interventions (Table 2; interventions 1, 2, 3, 6, 8–13, 15–17), one was a group intervention (Table 2, intervention 14), and three were combinations (Table 2; interventions 4, 5, 7). The choice of communication channel varied widely across interventions ranging from exclusively face-to-face communication (6 times) (Table 2; interventions 4, 7, 8, 11, 13, 14), mobile app (2 times) (Table 2; interventions 2, 3), messaging (4 times) (Table 2; interventions 10, 15, 16, 17), or a combination thereof (5 times) (Table 2; interventions 1, 5, 6, 9, 12). Nine interventions used additional materials for coaching, such as brochures/leaflets/workbooks (Table 2; interventions 1, 4, 6, 8, 9, 11–14), a website with written information (Table 2; interventions 10, 15), or an activity/step diary (Table 2; interventions 4, 13, 14). Additional voluntary interventions to increase workplace PA next to PA coaching were also integrated into some interventions. Examples were walking meetings (5 times) (Table 2; interventions 7–9, 16, 17), exercise sessions/PA programs (1 time) (Table 2, intervention 7), stair climbing (Table 2; interventions 16, 17), provision of a company bike and sit-stand desks (1 time) (Table 2, intervention 7), or a workstation treadmill and a video game system (2 times) (Table 2; interventions 16, 17).

**Fig. 2:**
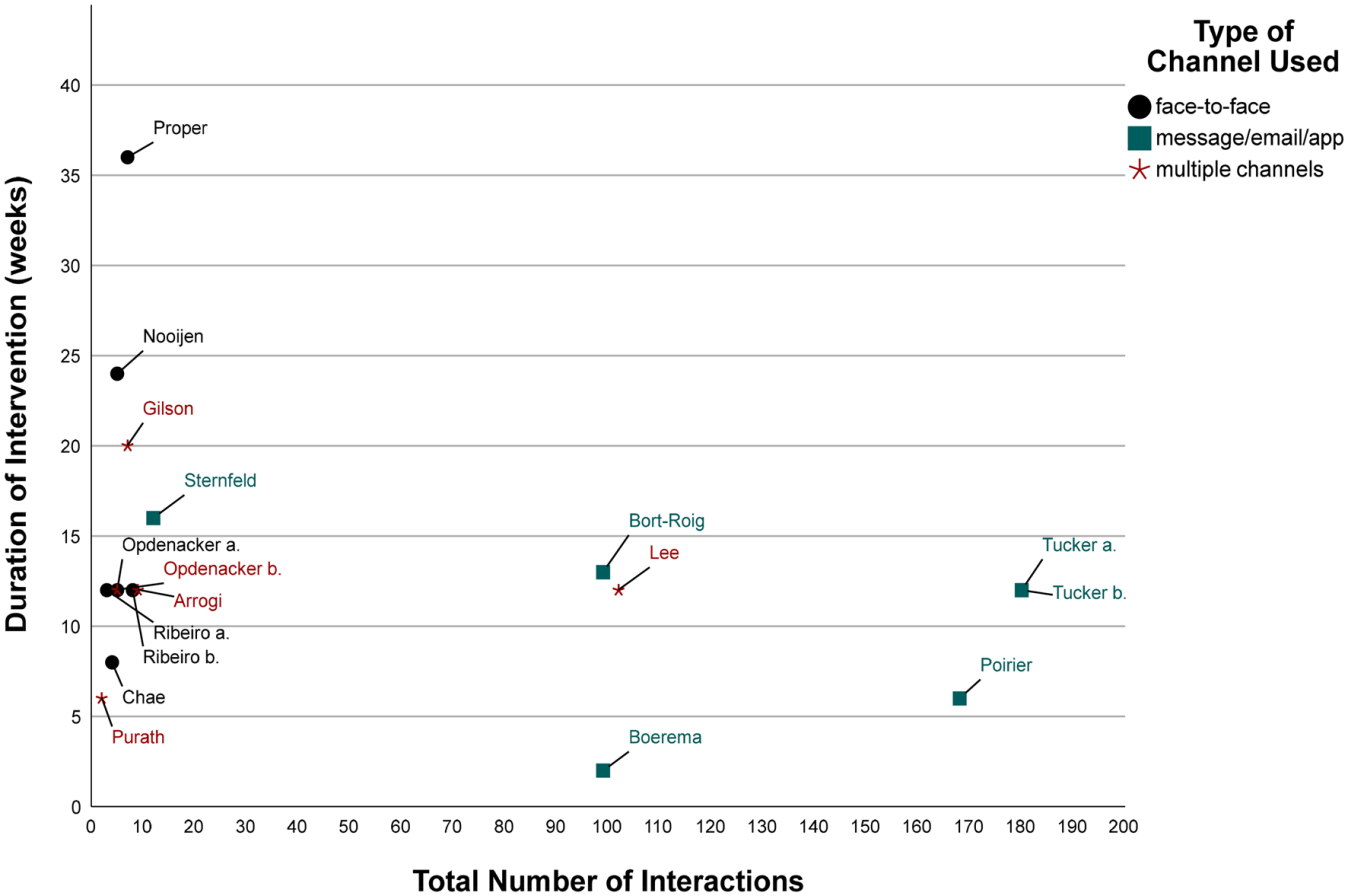
Overview of types of channels used, duration of interventions, and total number of interactions. Note. Total number of interactions = the highest number on the reported range of interactions between coach and coachee

Figure 2 presents an overview of the communication channels used in the interventions, the duration of the interventions, and the number of interactions

### Theory

Of the 17 interventions, five were based on a psychological theory (TTM: (Table 2; interventions 11, 12, 15), SCT: (Table 2, intervention 4), SDT: (Table 2, intervention 1).

### Behavior Change Techniques

Seventeen BCTs were identified throughout all interventions. The number of identified BCTs per intervention ranged from one to nine techniques. The six most frequently used techniques were goal setting (14 times), self-monitoring (of behavior and outcomes of behavior) (11 times), feedback (on behavior and outcomes of behavior) (9 times), information about health consequences (8 times), social support (7 times), and problem solving (7 times). The most frequently used combinations of BCTs were goal setting and self-monitoring (11 times), goal setting and information about health consequences (8 times), goal setting and feedback, goal setting and social support, and self-monitoring and feedback (6 times each) (Online Resource Table 4).

### Coach

The interventions used different types of coaches. They included coaching by personal contact with scientific personnel (6 times) (Table 2; interventions 1, 5, 8, 9, 16, 17) or health care personnel (6 times) (Table 2; interventions 4, 7, 11–14). Other four interventions relied on an e-coaching system (Table 2; interventions 2, 3, 6, 10). Two did not mention their type of coach [Table 2; interventions 6 (no information about face-to-face coach), 15)].

### Outcomes

PA outcomes as well as their measurements varied widely across all studies. Eight studies measured their PA outcomes (e.g., total energy expenditure, time spent walking or being active, numbers of flights of stairs and daily steps) objectively using different versions of accelerometers (Table 2; interventions 2, 3, 5, 7, 10, 16, 17) and pedometers (Table 2; interventions 4, 13, 14). Four studies used subjective measurement methods (questionnaires) (Table 2; interventions 8, 9, 11, 12, 15). The rest used a combination of both (Table 2; interventions 1, 6).

### Effects of Interventions on PA Outcomes

All 17 interventions indicated an increase in at least one PA outcome (Table 3). In terms of workplace PA, all but one intervention had an improvement in at least one workplace PA^3^ outcome (Table 3; interventions 1–8, 10–17). The intervention (Table 3, intervention 9) that did not yield an increase in workplace or total PA had an increase in leisure time PA only. Twelve of the 17 interventions indicated significant improvements in at least one workplace PA^3^ outcome after the intervention (Table 3; interventions 1, 3, 4, 6, 8, 10–16). Seven of these interventions showed a significant effect over time (Table 3; interventions 1, 3, 4, 6, 8, 11, 16), and eight showed a significant effect against another intervention group (Table 3; interventions 8, 14) or against a control group (Table 3; interventions 1, 6, 10, 12–15).

### Risk of Bias

**Fig. 3:**
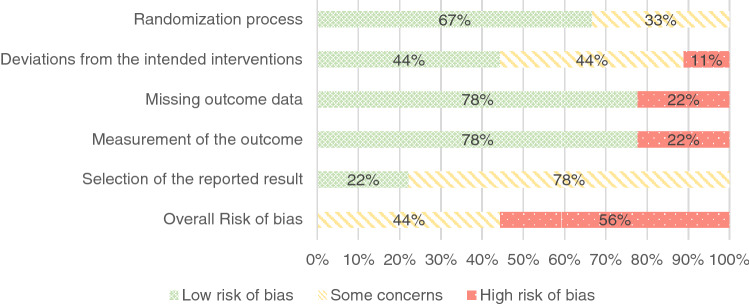
Overview of Risk of Bias of Randomized Controlled Studies by Domains

**Fig. 4:**
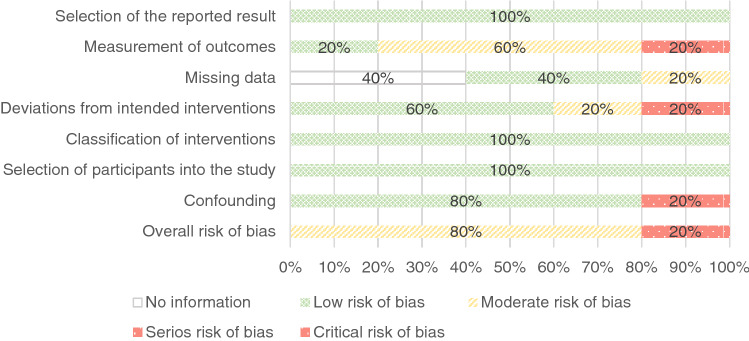
Overview of Risk of Bias of Non-Randomized Controlled Studies by Domains

After the initial assessment of risk of bias, the two raters who assessed study quality had an overall agreement on RoB 2 of 84.2% and on ROBINS-I of 92.9%. After a discussion meeting, there was agreement on all domains of the two instruments (Fig. 3 and 4).

Of nine RCTs assessed with the RoB 2 tool, five studies (Table 3; interventions 3, 10, 11, 12, 15) received a high risk of bias rating. The other four studies (Table 3; interventions 7–9, 13, 14, 16, 17) were rated as having some concerns. Study limitations and consequently a high risk of bias were due to deviations from intended interventions, missing outcome data, and the inappropriate measurement of outcomes.

Of five non-RCTs evaluated using the ROBINS-I tool, one (Table 3, intervention 1) received a critical risk of bias rating and the other four (Table 3; interventions 2, 4, 5, 6) a moderate risk of bias rating. Among the non-RCTs, the limitations of studies associated with a high risk of bias were due to deviations from intended interventions and due to confounding.

## Discussion

The aim of this review was to summarize previous workplace PA coaching interventions that aim to increase health-enhancing PA in the workplace. Included studies were longitudinal intervention studies. The intervention had to be PA coaching in the workplace, which had to be either web-/telephone-/mobile-based, or in person. The coaching had to include at least one interaction between coach and coachee, and it could either be individual or group coaching. The intervention had to aim at increasing health-enhancing PA in the workplace (plus optional leisure time PA, subjectively and/or objectively assessed) and be addressed toward sedentary employees. Thus, the present review provides an up-to-date overview of workplace PA coaching interventions, their compositions, and effectiveness.

As its main result, this review shows a wide variety of different compositions of PA coaching interventions in the workplace. These interventions varied greatly within the resources (e.g., organizational coaching parameters) and the activities (e.g., BCTs), thereby illustrating the diversity of PA coaching interventions implemented to date. In terms of outputs and outcomes, this review reveals that some studies have already used coaching in the workplace to increase PA. These studies showed positive effects on at least one PA outcome, but some showed only small changes. Overall, this review demonstrates the potential of PA coaching as a multifaceted workplace intervention with a high degree of individualization of parameters for different employee groups and different types of workplaces to increase PA.

In terms of the resources such as the organizational coaching parameters, this review found that a high variety of different communication channels (e.g., face-to-face/app/combinations) was integrated. Even though most studies substantiated their choice of communication channel, some did not provide any rationale (e.g., [51–53]). One good example are Poirier et al. [54], who substantiated their messaging intervention with benefits such as a great outreach at low cost and a “considerable public health impact” (p. 2). Other examples ([50]a, b.; [55]) integrated their e-coaching and PA into the work routines of employees to make the coaching as enjoyable and effective as possible. In order to understand the choice for a particular communication channel in a workplace PA coaching intervention, it is important that the rationale for this choice is also transparent to other researchers or practitioners.

Furthermore, it can be assumed that the resources communication channel and time scope parameters of coaching (e.g., the number of interactions) are interrelated, as, for example, coaching with an e-coach can provide "around-the-clock coaching" with many interactions in a shorter time period [45] in contrast to coaching with face-to-face contact. This "around-the-clock coaching" was found in interventions that used messaging, apps, or combinations thereof as communication channels (Fig. 2). However, e-coaching interventions based on messaging also eliminate the face-to-face contact that Wolever et al. [14] emphasize in their health coaching definition. The selection of the communication channel and consequently the number of interactions both depend on the specific coaching goals and organizational circumstances (e.g., the preferences and working routines of the target group, costs, or the technical possibilities).

In terms of activities, a variety of BCTs were used, but the reasons behind the choices for these techniques are crucial. Some researchers derived their BCTs theoretically or practically well. In terms of theory, SDT [51] and SCT [52] were used to derive BCTs. In terms of practical approaches, scholars [52, 56] used a practical community-based participatory research approach to engage the target population and/or other stakeholders. They identified and categorized the techniques needed to increase PA from the target population’s/stakeholders’ perspective. Other included interventions used pre-designed protocols/programs to develop their intervention, e.g., a PACE protocol (Patient-Centered Assessment and Counseling for Exercise protocol; Caparosa and Thompson 1999 in [57]) or the ALIVE program (A Lifestyle Intervention Via E-mail; Block et al. 2008 in [58]). In conclusion, there are different ways to justify integrated BCTs so far; however, there is no gold standard yet. Whether concept developers take the practical, theoretical or empirical route to justifying BCTs, it is particularly important to consider the target groups and individuals in the final developmental step.

It is difficult to draw conclusions about which BCTs and which combinations of BCTs are most effective due to the diversity as well as the interrelation of the different coaching parameters within the different interventions. This is consistent with a recent meta-review by Spring et al. [59], who similarly found little evidence for the isolated effectiveness of single BCTs in promoting PA, because there was no evidence for one BCT evaluated in more than one meta-analysis whose inclusion in PA interventions was associated with better outcomes.

Moreover, in some included studies—in addition to coaching—other activities such as organized voluntary interventions were implemented to increase workplace PA. Examples of these interventions include bike routes around campus ([60] a. & b.), treadmills in the workplace ([50] a. & b.), and sports promoters in departments [53, 60] a. & b.]. These interventions demonstrate that PA can be integrated well into the workplace. To conclude, the implementation of additional voluntary interventions can facilitate the increase in physical activity targeted by coaching and should always be considered in coaching concepts.

In addition to these voluntary workplace interventions, some interventions also integrated additional techniques to increase PA during leisure time, e.g., techniques that aim at increasing PA in the active transportation context or leisure context [58, 61] a. & b.]. Thus, workplace PA coaching ideally targets PA in all contexts, such as leisure and active transportation, so that a transfer effect can take place and a PA increase is present in each context.

Lastly, the included studies showed a great outreach to people of many age groups (up to retirement), different genders, sectors, and social classes. In this review, among others, employees of health care companies, pharmacies, but also universities, airlines, and ambulatory clinic services are represented. This overcomes the barrier to PA-enhancing interventions of limited accessibility to different groups of people [18] and shows that workplace PA coaching can address many different groups of people. However, groups of people that are typically not included in workplace PA coaching are unemployed persons, pensioners, self-employed persons, children, and young adolescents. Future reflection and research should therefore address workplace-like settings in which PA coaching can be integrated to reach these non-working target groups.

Although the included studies could not be meta-analyzed due to their wide diversity, they appear to have a positive impact on workplace PA. With the exception of one intervention, all interventions showed either positive effects for at least one workplace or total PA outcome (e.g., active transportation, total energy expenditure, or weekday step count) either over time or compared to other active or passive control groups. This output supports the evidence that coaching interventions are able to increase PA in sedentary employees just as in other target groups [40, 41]. Moreover, another output is acceptability of the included interventions that has been shown through relatively high response rates (*M* = 77%) which indicate high adherences of the target groups for intervention studies. Reasons for these high adherence rates could be, first, the interactivity, individuality, and person-centeredness that characterize the coaching interventions included here [14], and second, the many different coaching parameters described, each of which can be modified to suit the occupational target group.

### Limitations

Limitations of this review relate primarily to the difficulty of comparing the included interventions. This difficulty has also been acknowledged in other reviews (e.g., [14]) and can mostly be attributed to two main issues: 1) *the different goals of the interventions;* and 2) *the varying intervention descriptions*.

The different goals of the interventions are problematic because they lead to different intervention implementations and different BCTs, making their comparability difficult. Some interventions only aim at evaluating PA coaching and related PA behavior, while other interventions additionally evaluate other behaviors, such as sedentary or dietary behavior or the improvement of parameters such as fat mass. In terms of PA and sedentary behavior, it can further be discussed to what extent there is a difference between increasing PA and decreasing sedentary behavior, because decreasing sedentary behavior actually also aims at increasing PA.

The varying intervention descriptions further complicate the comparison of the different interventions. In particular, some intervention descriptions [51, 54–56, 58, 62, 63] were formulated in a detailed and comprehensible manner. Inadequate intervention descriptions make replication studies infeasible and do not add much value to practitioners or other researchers. In some cases, intervention descriptions were not described properly and so inadequate that only one BCT could be detected and inferred (e.g., [57]), whereas others were detailed enough so that up to 9 BCTs could be identified [56].

In addition to the limitations concerning the intervention level, there are also some limitations that regard the study design, as the risk of bias results show. First, there were deviations from intended interventions, as both participants (in this case, coachees) and carers/people delivering the interventions (in this case, coaches) could not be blinded to a coaching intervention. Second, most study publications did not provide evidence that the results were not biased by missing outcome data. Third, some study results are limited due to problematic outcome measures, such as less feasible accelerometers (incorrect carrying position on body; [62]) or mere subjective measurement of PA ([60] a. & b.; [57, 58, 64]).

Two limitations that generally apply to reviews must also be considered when interpreting the results. First, our literature search was restricted to academic articles published in English and German. This may have resulted in the exclusion of relevant studies published in other languages or in gray literature sources. Second, there is a risk for publication bias, as interventions that yield a negative or insignificant outcome are less likely to be published [65].

### Strengths

The strengths of this review are, on the one hand, the provision of the first general overview of the state of the literature on workplace PA coaching to increase PA in the workplace. Second, the Cochrane tools RoB 2 [48] and ROBINS-I [49] were used to assess the risk of bias. These are validated but challenging to implement even for raters with extensive experience [66, 67]. A third strength regards the high interrater reliability between the two raters of the studies (RoB 2: 84.2%, ROBINS-I: 92.9%).

### Recommendations for Practice

Two features for recommendations for practice are particularly noteworthy when considering, in particular, those studies that show significant effects against a control group or another intervention group. The first feature of these studies is a combination of BCTs, more specifically goal setting, self-monitoring, and problem solving. These techniques have both a motivational, as well as a volitional focus, and therefore form a good basis for health behavior change [68]. The second feature concerns the communication channel. Face-to-face coaching alone or in combination with messaging, phone calls or an app should take precedence over digital-only coaching. Face-to-face enables a more intensive personal relationship, which Wolever et al. [14] already emphasized in their coaching definition.

### Recommendations for Future Research

Some included studies provided inadequate intervention descriptions, making the actual coaching difficult to understand or replicate. A possible approach would be to use a unified language for BCTs as tools for coaching programs [69]. One possibility for a collection of such techniques would be the taxonomy by Michie et al. [32], which was developed in a Delphi survey with behavior change experts.

In addition to the insufficient intervention descriptions, many derivations of the interventions were not comprehensible. It is useful for both other researchers and practitioners to comprehend why specific techniques were used and combined with others to understand the theoretical mechanisms underlying the effects. One approach is to derive and combine different BCTs according to health behavior change theories (HAPA; [70]) by implementing motivational techniques (e.g., goal setting) to form the intention and volitional techniques (e.g., action planning) in order to support the implementation of this intention. As a second approach, some studies used a community-based participatory research approach with the help of group conversations (e.g., [56]) to develop the coaching intervention based on a needs assessment prospectively. Both approaches could be used in future coaching interventions to allow for the development of a coaching intervention that is tailored to the needs and desires of the participants.

Only a few included studies integrated a follow-up phase with additional measurements. These additional phases and measurements are important in order to evaluate the long-term effectiveness of future coaching interventions because only long-term intervention effects show lasting success.

The individual interventions included considered coaching resources (e.g., organizational coaching parameters), coaching activities (e.g., BCTs) and coaching outputs (increasing PA), but there is little information on the outcomes of the interventions, such as the psychological and physical improvements of increased physical activity behavior on well-being or job satisfaction. In this review, only one study [63] examined worker's productivity as an outcome. Future research should therefore also consider and co-evaluate the short-, medium-, and long-term effects of the interventions.

## Conclusion

In conclusion, this overview shows that coaching has already been used in some studies to increase the PA of employees, all with positive effects in at least one variable, whereby some only led to low changes in certain variables. This paper is the first review to provide an overview of the current state of the literature on workplace PA coaching and shows a range of different coaching interventions and their compositions, including different resources (e.g., organizational coaching parameters) and activities (e.g., BCTs). Due to the resulting different coaching approaches, where each parameter can be changed individually, PA coaching in the workplace can contribute to the improvement of employees' PA. The workplace could thus become another coaching setting besides schools, health care organizations or community centers to counteract the lack of PA in everyone's daily life.

## Supplementary Information

Below is the link to the electronic supplementary material.

Electronic supplementary material 1 (DOCX 21 kb)

## Data Availability

The data supporting this systematic review are from previously reported studies, which have been cited. The processed data are available from the corresponding author on reasonable request.

## Footnotes

1 Some extracted data categories have different terms in the registered protocol. To report the data, more global terms were used in some cases because the data were more heterogeneous than initially thought (occupational group became professional sector, coaching sessions became coaching interactions). In other cases, more precise terms were used for a better understanding (other potential components addressed in coaching became other behaviors potentially addressed in coaching, medium of intervention became communication channel, and type of BCT became name of BCT, transport PA became active transportation).

2 The 14 included studies resulted in 17 interventions, since Opdenacker and Boen [60], Ribeiro et al. [61] ), and Tucker et al. [50] each included two intervention groups (e.g. [50]; a. & b.) relevant for this review. When we talk about *Sample Characteristics*, *Study Design, Outcomes* and *Risk of Bias*, we are referring to studies (but list the interventions in the parentheses). In all other paragraphs in the results section, we talk about the 17 interventions.

3 In some interventions, workplace PA is measured separately; in other interventions, it is measured as part of the total PA outcomes.
