# Supplementary material for: A Systematic Review of Workplace Physical Activity Coaching
Source: J Occup Rehabil. 2023 Feb 27;33(3):550–69. doi: 10.1007/s10926-023-10093-8 (PMC10495277; doi:10.1007/s10926-023-10093-8)
Supplement: Supplementary file 1 — Electronic supplementary material 1 (DOCX 21 kb) [file 10926_2023_10093_MOESM1_ESM.docx]

|  | Goal setting | Self-monitoring | Feedback | Information about health consequences | Social support | Problem solving | Action planning | Prompts/cues | Behavior substitution | Reward/ incentive | Monitoring of outcome(s) of behavior without feedback | Habit formation | Adding objects to the environment | Biofeedback | Discrepancy between current behavior and goal | Behavioral contract | Instruction on how to perform the behavior |
| --- | --- | --- | --- | --- | --- | --- | --- | --- | --- | --- | --- | --- | --- | --- | --- | --- | --- |
| Goal setting |  | 9 | 7 | 8 | 7 | 6 | 3 | - | 2 | 2 | 2 | 1 | 1 | 1 | 1 | 1 | 1 |
| Self-monitoring |  |  | 6 | 6 |  | 6 |  | 1 | 3 | 2 | - | 1 | 1 | 1 | 1 | - | 1 |
| Feedback |  |  |  | 3 | 4 | 3 | 3 | 2 | 2 | 2 | - | 1 | 1 | 1 | - | 1 | - |
| Information about health consequences |  |  |  |  | 4 | 4 | 1 | - | 2 | 1 | - | 1 | 1 | - | 1 | 1 | 1 |
| Social support |  |  |  |  |  | 4 | 1 | - | - | 1 | - | - | - | - | - | - | - |
| Problem solving |  |  |  |  |  |  | 1 | - | 1 | - | - | - | - | 1 | 1 | 1 | - |
| Action planning |  |  |  |  |  |  |  | - | 1 | 1 | - | 1 | 1 | 1 | - | - | - |
| Prompts/cues |  |  |  |  |  |  |  |  | 1 | - | - | - | - | 1 | - | - | - |
| Behavior substitution |  |  |  |  |  |  |  |  |  | 1 | - | 1 | 1 | - | 1 | - | - |
| Reward/ incentive |  |  |  |  |  |  |  |  |  |  | - | 1 | 1 | - | - | - | - |
| Monitoring of outcome(s) of behavior without feedback |  |  |  |  |  |  |  |  |  |  |  | - | - | - | - | - | - |
| Habit formation |  |  |  |  |  |  |  |  |  |  |  |  | 1 | - | - | - | - |
| Adding objects to the environment |  |  |  |  |  |  |  |  |  |  |  |  |  | - | - | - | - |
| Biofeedback |  |  |  |  |  |  |  |  |  |  |  |  |  |  | - | - | - |
| Discrepancy between current behavior and goal |  |  |  |  |  |  |  |  |  |  |  |  |  |  |  | - | - |
| Behavioral contract |  |  |  |  |  |  |  |  |  |  |  |  |  |  |  |  | - |
| Instruction on how to perform the behavior |  |  |  |  |  |  |  |  |  |  |  |  |  |  |  |  |  |

International Archives of Occupational and Environmental Health

A Systematic Review of Workplace Physical Activity Coaching

A. Gawlik^1^, J. Lüdemann^1^, A. Neuhausen^2^, C. Zepp^1^, F. Vitinius^2^ & J. Kleinert^1^

^1^ Institute of Psychology, German Sport University Cologne, Cologne, Germany, ^2^ Department of Psychosomatics and Psychotherapy, University of Cologne, Cologne, Germany

Corresponding author: [a.gawlik@dshs-koeln.de](mailto:a.gawlik@dshs-koeln.de)

**Online Resource**

**Tab. 4** Number of combinations of BCTs in included interventions
